# Preventing Cardiac Damage in Patients Treated for Breast Cancer and Lymphoma: The PROACT Clinical Trial

**DOI:** 10.1016/j.jaccao.2024.07.010

**Published:** 2024-08-27

**Authors:** David Austin, Rebecca H. Maier, Nasima Akhter, Mohammad Sayari, Emmanuel Ogundimu, Jamie M. Maddox, Sharareh Vahabi, Alison C. Humphreys, Janine Graham, Helen Oxenham, Sophie Haney, Nicola Cresti, Mark Verrill, Wendy Osborne, Kathryn L. Wright, Rebecca Goranova, James R. Bailey, Nagesh Kalakonda, Mac Macheta, Mari F. Kilner, Moya E. Young, Nick J. Morley, Pratap Neelakantan, Georgia Gilbert, Byju K. Thomas, Richard J. Graham, Takeshi Fujisawa, Nicholas L. Mills, Victoria Hildreth, Jonathan Prichard, Adetayo S. Kasim, Helen C. Hancock, Chris Plummer

**Affiliations:** a Academic Cardiovascular Unit, James Cook University Hospital, South Tees Hospitals NHS Foundation Trust, Middlesbrough, United Kingdom; b Population Health Sciences Institute, Newcastle University, Newcastle upon Tyne, United Kingdom; c School of Health and Life Sciences, Teesside University, Middlesbrough, United Kingdom; d Department of Mathematical Science, Durham University, Durham, United Kingdom; e Department of Haematology, South Tees Hospitals NHS Foundation Trust, Middlesbrough, United Kingdom; f Bristol Heart Institute, University Hospitals Bristol and Weston NHS Foundation Trust, Bristol, United Kingdom; g Cancer Services, South Tees Hospitals NHS Foundation Trust, Middlesbrough, United Kingdom; h Cancer Services, North Tees and Hartlepool NHS Foundation Trust, Stockton-on-Tees, United Kingdom; i Department of Cardiology, North Tees and Hartlepool NHS Foundation Trust, Stockton-on-Tees, United Kingdom; j Cancer Services, County Durham and Darlington NHS Foundation Trust, Darlington, United Kingdom; k Freeman Hospital, Newcastle upon Tyne Hospitals NHS Foundation Trust, Newcastle upon Tyne, United Kingdom; l Cancer Services, South Tyneside and Sunderland NHS Foundation Trust, Sunderland, United Kingdom; m Department of Oncology, Derriford Hospital, University Hospitals Plymouth NHS Trust, Plymouth, United Kingdom; n Department of Haematology, Hull University Teaching Hospitals NHS Trust, Queen’s Centre for Oncology and Haematology, Castle Hill Hospital, Hull, United Kingdom; o Department of Molecular and Clinical Cancer Medicine, University of Liverpool, Liverpool, United Kingdom; p Department of Haematology, Blackpool Teaching Hospitals, Blackpool, United Kingdom; q Pathology Services, Northumbria Healthcare NHS Foundation Trust, North Shields, United Kingdom; r Department of Haematology, East Kent Hospitals University NHS Foundation Trust, Canterbury, United Kingdom; s Department of Haematology, Sheffield Teaching Hospitals NHS Foundation Trust, Sheffield, United Kingdom; t Department of Haematology, Royal Berkshire NHS Foundation Trust, Reading, United Kingdom; u Centre for Cardiovascular Science, University of Edinburgh, Edinburgh, United Kingdom; v Newcastle Clinical Trials Unit, Newcastle University, Newcastle upon Tyne, United Kingdom; w Department of Anthropology, Durham University, Durham, United Kingdom; x Department of Cardiology, Newcastle upon Tyne Hospitals NHS Foundation Trust, Newcastle upon Tyne, United Kingdom; y Faculty of Medical Sciences, Newcastle University, Newcastle upon Tyne, United Kingdom

**Keywords:** anthracycline, biomarkers, breast cancer, echocardiography, lymphoma, prevention

## Abstract

**Background:**

Cardiotoxicity is a concern for cancer survivors undergoing anthracycline chemotherapy. Enalapril has been explored for its potential to mitigate cardiotoxicity in cancer patients. The dose-dependent cardiotoxicity effects of anthracyclines can be detected early through the biomarker cardiac troponin.

**Objectives:**

The PROACT (Preventing Cardiac Damage in Patients Treated for Breast Cancer and Lymphoma) clinical trial assessed the effectiveness of enalapril in preventing cardiotoxicity, manifesting as myocardial injury and cardiac function impairment, in patients undergoing high-dose anthracycline-based chemotherapy for breast cancer or non-Hodgkin lymphoma.

**Methods:**

This prospective, multicenter, open-label, randomized controlled trial employed a superiority design with observer-blinded endpoints. A total of 111 participants, scheduled for 6 cycles of chemotherapy with a planned dose of ≥300 mg/m^2^ doxorubicin equivalents, were randomized to receive either enalapril (titrated up to 20 mg daily) or standard care without enalapril.

**Results:**

Myocardial injury, indicated by cardiac troponin T (≥14 ng/L), during and 1 month after chemotherapy, was observed in 42 (77.8%) of 54 patients in the enalapril group vs 45 (83.3%) of 54 patients in the standard care group (OR: 0.65; 95% CI: 0.23-1.78). Injury detected by cardiac troponin I (>26.2 ng/L) occurred in 25 (47.2%) of 53 patients on enalapril compared with 24 (45.3%) of 53 in standard care (OR: 1.10; 95% CI: 0.50-2.38). A relative decline of more than 15% from baseline in left ventricular global longitudinal strain was observed in 10 (21.3%) of 47 patients on enalapril and 9 (21.9%) of 41 in standard care (OR: 0.95; 95% CI: 0.33-2.74). An absolute decline of >10% to <50% in left ventricular ejection fraction was seen in 2 (4.1%) of 49 patients on enalapril vs none in patients in standard care.

**Conclusions:**

Adding enalapril to standard care during chemotherapy did not prevent cardiotoxicity in patients receiving high-dose anthracycline-based chemotherapy. (PROACT: Can we prevent Chemotherapy-related Heart Damage in Patients With Breast Cancer and Lymphoma?; NCT03265574)

Anthracyclines are highly effective in treating various cancers, including breast cancer and hematologic malignancies. Despite their efficacy, these drugs can cause myocardial injury that leads to impaired cardiac function and heart failure.^1^, ^2^, ^3^ Thus, preventing anthracycline-induced cardiotoxicity is critical for reducing the cardiovascular risks in the growing population of cancer survivors.^4^, ^5^, ^6^

Anthracycline cardiotoxicity is dose dependent and referred to as cancer therapy–related cardiac dysfunction (CTRCD). Cardiac troponin (cTn), an early marker, plays a pivotal role in detecting cardiotoxicity.^6^, ^7^, ^8^ Studies indicate that a normal or undetectable cTn level during or 1 month after anthracycline treatment correlates with a low risk of significant cardiotoxicity.^9^ Conversely, elevated cTn levels during or after treatment are associated with increasing rates of subsequent cardiotoxicity. Notably, 1 clinical trial demonstrated that using the angiotensin-converting enzyme inhibitor enalapril normalized elevated cTn levels and prevented early declines in left ventricular (LV) function.^10^ This protective effect, supported by animal studies and other small clinical trials, highlights the potential of angiotensin-converting enzyme inhibitors in the context of anthracycline chemotherapy.^11^, ^12^, ^13^, ^14^, ^15^, ^16^, ^17^

The PROACT (Preventing Cardiac Damage in Patients Treated for Breast Cancer and Lymphoma) trial aims to evaluate the effectiveness of enalapril in preventing cardiotoxicity among patients with breast cancer and non-Hodgkin lymphoma (NHL) undergoing high-dose (≥300 mg/m^2^ doxorubicin equivalents)^18^ anthracycline-based chemotherapy.

## Methods

### Study Design

The PROACT trial was a prospective, multicenter, open-label, randomized controlled trial utilizing a superiority design with observer-blinded endpoints. It evaluated the effectiveness of enalapril (intervention) in preventing cardiotoxicity compared with standard care (no enalapril, comparator) in patients with breast cancer or NHL. Participants were scheduled for 6 cycles of high-dose anthracycline-based chemotherapy. Details of the trial design and protocol have been documented in previous publications,^17^ and the final approved statistical analysis plan is available in the Supplemental Appendix.

The PROACT trial enrolled patients from 13 sites across the United Kingdom, under the supervision of a trial management group that included the trial sponsor. Oversight was ensured by independent data monitoring and trial steering committees, which included patient representatives and met regularly. Additional details are available in the Supplemental Appendix. The study was funded by the National Institute for Health and Care Research, under the Research for Patient Benefit program. Ethical approval was obtained from the NHS West Midlands Edgbaston Research Ethics Committee (17/WM/0248), and all participants provided written informed consent.

### Participants

Adults scheduled to undergo 6 cycles of anthracycline-based chemotherapy for histopathologically confirmed breast cancer (after surgery) or NHL were eligible to participate. The total planned anthracycline dose was ≥300 mg/m^2^ doxorubicin equivalents. Permissible breast cancer regimens included epirubicin and cyclophosphamide (EC90) (with 540 mg mg/m^2^ epirubicin or 432 mg/m^2^ doxorubicin equivalents) and fluorouracil, epirubicin, and cyclophosphamide (with 450 mg/m^2^ epirubicin or 360 mg/m^2^ doxorubicin equivalents). The NHL regimen included cyclophosphamide, doxorubicin, vincristine, and prednisolone ± rituximab (with 300 mg/m^2^ doxorubicin). Participants with HER2-positive breast cancer were included if trastuzumab was scheduled to begin after the final primary endpoint assessment.

Individuals were excluded if they had baseline myocardial injury (cardiac troponin T [cTnT] concentration of ≥14 ng/L), left ventricular ejection fraction (LVEF) <50%, contraindications to enalapril, or current use of renin-angiotensin-aldosterone system (RAAS) inhibitors. Detailed inclusion and exclusion criteria are provided in the Supplemental Appendix.

### Randomization and Blinding

Consenting and eligible participants were randomized in a 1:1 ratio to receive either enalapril or standard care. This randomization was managed using a central, secure, 24-hour Web-based system with concealed allocation. A minimization strategy accounted for the planned 6-cycle chemotherapy regimen, and for breast cancer patients, HER2 status. Although the trial was open label, primary and secondary outcomes were independently assessed by biochemistry and echocardiographic core laboratories, which remained blinded to participant allocation. Additional details are available in the Supplemental Appendix.

### Intervention

Participants assigned to enalapril started treatment at least 2 days before chemotherapy began. The initial dose was 2.5 mg twice daily, with 2 subsequent titration visits aimed at incrementally adjusting the dose based on blood pressure, biochemistry results, and side effects. The target was to achieve a dosage of 10 mg twice daily (20 mg/d), with adjustments for maximum tolerated dose made at the clinician’s discretion. Final dose adjustments were allowed within the first cycle of chemotherapy to prevent any treatment delays. Participants continued enalapril throughout their chemotherapy regimen, including during any treatment delays, until 3 weeks after the final anthracycline dose. Temporary dose reductions or a single temporary discontinuation was permitted based on clinical judgement.

### Outcomes

The primary outcome was myocardial injury, defined by the presence (≥14 ng/L) or absence (<14 ng/L) of cTnT elevation. cTnT levels were measured prior to each chemotherapy cycle (<72 hours before each dose) or 1 month after the final anthracycline dose using a highly sensitive Elecsys assay (Roche) on heparinized plasma. Measurements were conducted in 2 batched runs, with the assay demonstrating an interassay coefficient of variation <10% at the upper limit of normal (ULN) of <14.0 ng/L and a lower detection limit of 5 ng/L.^19^

As a secondary outcome, myocardial injury was assessed using a cardiac troponin I (cTnI) assay. cTnI was measured on heparinized plasma in a single batch using the ARCHITECT_*STAT*_ high-sensitivity cTnI assay (Abbott Laboratories). This assay features a detection limit of 1.2 ng/L and an interassay coefficient of variation <10% at 4.7 ng/L, with an upper reference limit of 26.2 ng/L.^20^

Secondary outcomes related to cardiac function were assessed via transthoracic echocardiography at baseline and 1 month after chemotherapy. These assessments focused on absolute and relative changes in LV global longitudinal strain (GLS) and a binary endpoint of a relative decline >15% from baseline. For LVEF, the secondary outcomes considered changes from baseline and a binary endpoint of an absolute decline >10% to an LVEF <50%. Echocardiography was performed locally by recruiting teams and centrally reported for trial outcomes by experienced echocardiographers (S.V., B.K.T., R.J.G.), who were blinded to treatment allocation, using vendor-independent software (TOMTEC; Philips).

The safety of enalapril was monitored by documenting adverse reactions and both adverse and serious adverse events throughout the trial. Additionally, cancer and cardiovascular outcomes were recorded for the study population. Cardiotoxicity was defined in accordance with the current UK echocardiography guidelines^21^ and the European Society of Cardiology cardio-oncology guideline criteria.^6^

### Statistical Analysis

The initial sample size calculation was based on detecting a reduction in myocardial injury incidence from 47% to 20%. To achieve 90% statistical power with a 2-sided Fisher exact test, 140 patients (70 in each group) were needed. Due to recruitment challenges, the sample size was revised in agreement with the funder. With the same endpoints but adjusted for 80% power, the required number of patients was recalculated to 106 to detect a reduction in the proportion of patients with cTnT present from 47% to 20%. A detailed description of the sample size calculation is available in previously published material.^17^

Continuous data are summarized by study group using mean ± SD for normally distributed data and median (Q1-Q3) for skewed data. Categorical data are presented as frequency and percentage. The primary outcome analysis adhered to the modified intention-to-treat principle, including all randomized patients who had contributed data. Logistic regression, adjusted for the minimization factor—chemotherapy regimen—was used to analyze the primary outcome. Site clustering was not considered due to the standardized chemotherapy protocols and centralized cTnT analysis. Treatment effects are expressed as ORs with 95% CIs, and a *P* value of <0.05 was considered statistically significant. No imputation was made for missing data.

Secondary binary outcomes were analyzed under the modified intention-to-treat principle using logistic regression for all available data. Firth logistic regression was applied in situations involving empty or small cell counts.^22^ No adjustments were made for the type I error rate across multiple testing of secondary endpoints; thus, these are considered exploratory, and the reported 95% CIs were not adjusted for multiplicity. Sensitivity analyses were performed for both per-protocol and as-treated populations. All statistical analyses were performed using R version 4.3.1 (R Foundation for Statistical Computing) and IBM SPSS Statistics version 27.0.

## Results

### Study Population

**Figure 1:**
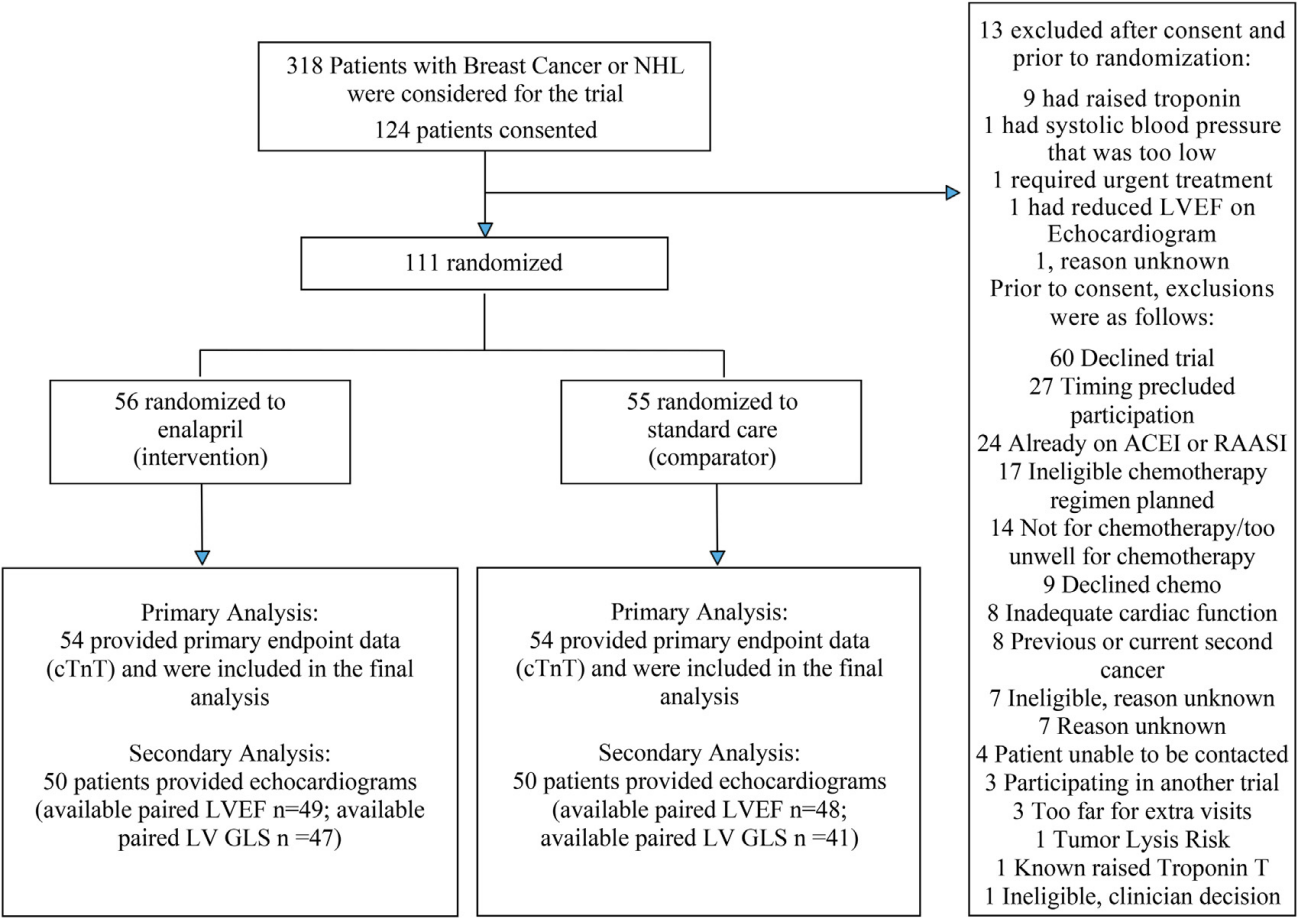
Participant Flow Throughout the PROACT Trial This figure presents a CONSORT diagram outlining the flow of participants throughout the PROACT (Preventing Cardiac Damage in Patients Treated for Breast Cancer and Lymphoma) trial. (Top) All patients initially considered for inclusion; (center) the progression to randomization. (Right) Reasons for exclusion both before and after obtaining informed consent. (Bottom) The patients who were included in the analyses for primary and secondary endpoint analyses. ACEI = angiotensin-converting enzyme inhibitor; cTnT = cardiac troponin T; GLS = global longitudinal strain; LV = left ventricular; LVEF = left ventricular ejection fraction; NHL = non-Hodgkin lymphoma; RAAS = renin-angiotensin-aldosterone system inhibitor.

Of the 318 patients identified, 124 consented, and 111 were ultimately randomized between October 2017 and March 2023. The participants were split into 2 groups: 56 patients in the enalapril group (intervention) and 55 in standard care (comparator). The main reason for exclusion after consent was an elevated baseline cTnT level, particularly in patients with NHL (Figure 1). Within the intervention group, 2 patients withdrew early, and 1 patient in the standard care group was unable to start chemotherapy due to the onset of the COVID 19 pandemic.

At baseline, the 2 groups were comparable (Table 1), though a higher incidence of hypertension was observed in the enalapril group. The mean age of participants was 58 ± 11 years, with 86 (77.5%) being female. Of the participants, 107 (96.4%) self-reported their ethnicity as White British. Breast cancer was present in 69 (62.2%) participants, while 42 (37.8%) had NHL. The most common primary diagnoses were ductal breast cancer and diffuse large B cell lymphoma, detailed in Supplemental Table 1. Treatment regimens were similar across study groups; in the breast cancer subset, 17 (24.6%) participants were on the fluorouracil, epirubicin, and cyclophosphamide regimen and 52 (75.4%) on the EC90 regimen. All NHL patients received cyclophosphamide, doxorubicin, vincristine, and prednisolone ± rituximab.

**Table 1:** Baseline Characteristics of the Study Participants

	Enalapril (n = 56)	Standard care (n = 55)
Demographic		
Age at randomization, y	58 ± 11	58 ± 12
Sex		
Female	45 (80.4)	41 (74.5)
Male	11 (19.6)	14 (25.5)
Race^a^		
White	55 (98.2)	52 (94.5)
Other	1 (1.8)	3 (5.5)
Body mass index, kg/m^2^	28.3 (4.8)	28.2 (5.5)
Type of cancer		
Breast cancer	35 (62.5)	34 (61.8)
Non-Hodgkin lymphoma	21 (37.5)	21 (38.2)
Clinical history		
Atrial fibrillation	1 (1.8)	1 (1.8)
ECOG performance status scale^b^		
Grade 0	49 (87.5)	48 (87.3)
Grade 1	6 (10.7)	7 (12.7)
Grade 2	1 (1.8)	0 (0.0)
Coronary heart disease	2 (3.6)	2 (3.6)
Diabetes	5 (8.9)	3 (5.5)
Hypertension	12 (21.4)	5 (9.1)
Hyperlipidemia	5 (8.9)	3 (5.5)
Smoking		
Current smoker	7 (12.5)	3 (5.5)
Ex-smoker	22 (39.3)	15 (27.3)
Never smoked	27 (48.2)	37 (67.3)
HFA/ICOS risk^c^		
Low	29 (52.7)	22 (42.9)
Medium	23 (41.8)	22 (39.3)
High	3 (5.5)	10 (17.7)
Baseline clinical assessments		
Heart rate, beats/min	74.7 ± 10.8	75.8 ± 10.5
Systolic blood pressure, mm Hg	132.8 ± 13.9	135.8 ± 15.5
Diastolic blood pressure, mm Hg	80.7 ± 9.9	80.4 ± 8.6
Creatinine, μmol/L	65.0 ± 12.3	67.5 ± 11.4
Statin therapy, n (%)	4 (7.1)	6 (10.9)
Planned chemotherapy regimen		
FEC75	8 (14.3)	9 (16.4)
EC90	27 (48.2)	25 (45.5)
(R-)CHOP	21 (37.5)	21 (38.2)
Chest radiotherapy prior to chemotherapy		
Both sides	1 (1.8)	1 (1.8)
Left side	0 (0.0)	1 (1.8)
Right side	1 (1.8)	2 (3.8)
Not known	0 (0.0)	1 (1.8)
None	54 (96.4)	50 (90.9)

Values are mean ± SD or n (%).

EC90 = epirubicin and cyclophosphamide; ECOG = Eastern Cooperative Oncology Group; FEC75 = fluorouracil, epirubicin and cyclophosphamide; HFA/ICOS = Heart Failure Association/International Cardio-Oncology Society; (R)-CHOP = cyclophosphamide, doxorubicin, vincristine and prednisolone (± rituximab).

a Race was self-reported.

b ECOG performance status was based on the following grades: 0 = fully active able to carry on all predisease performance without restriction; 1 = restricted in physically strenuous activity but ambulatory and able to carry out work of a light or sedentary nature (eg, light housework, office work); 2 = ambulatory and capable of all self-care but unable to carry out any work activities; Up and about more than 50% of waking hours.

c HFA/ICOS risk was calculated retrospectively.

The chemotherapy regimens and received anthracycline doses were similar across study groups. In the enalapril group, the mean anthracycline dose was 323 ± 96 mg/m^2^ doxorubicin equivalents, compared with 334 ± 100 mg/m^2^ doxorubicin equivalents in the standard care group. Regarding enalapril dosing, 12 (23%) patients in the enalapril group were titrated to 5 mg twice daily, while the remaining 41 (77%) reached a dose of 10 mg twice daily. The average daily titrated dose was 17.7 ± 4.2 mg (Table 2). Notably, the enalapril group exhibited a marked reduction in systolic blood pressure, with a decrease >10 mm Hg by the end of treatment (Supplemental Figure 1).

**Table 2:** Chemotherapy and Enalapril Treatment

	Enalapril (n = 56)	Standard care (n = 55)
Chemotherapy cycles received		
Mean ± SD	5.3 ± 1.4	5.5 ± 1.4
Median (range)	6 (0-6)	6 (0-6)
Anthracycline dose Received, mg/m^2^,^a^		
Mean ± SD	323 ± 96	334 ± 100
Maximum titrated daily enalapril dose, mg		
Mean ± SD	17.7 ± 4.2	N/A
Median (range)	20 (10-20)	N/A

N/A = not applicable.

a Doxorubicin-equivalent dose (mg/m^2^).

### Primary Outcome

Myocardial injury, observed either during or 1 month after anthracycline chemotherapy, occurred in 78.8% (n = 42 of 54) of patients in the enalapril group and 83.3% (n = 45 of 54) in the standard care group. No significant difference was found between enalapril and standard care when adjusted for chemotherapy regimen, with an OR of 0.65 (95% CI: 0.23 to 1.78; *P* = 0.41) (Table 3). These results remained consistent across both per-protocol and as-treated sensitivity analyses (Supplemental Figure 2).

**Table 3:** Logistic Regression Results for Primary and Secondary Outcomes

	Indicator	Group	Total	Adjusted OR (95% CI)^a^	*P* Value
Primary^a^					
	cTnT	Enalapril	42/54 (77.7)	0.65 (0.23-1.78)^b^	0.41^c^
		Standard care	45/54 (83.3)		
Secondary^a^					
	cTnI	Enalapril	25/53 (47.2)	1.10 (0.50-2.38)	0.82^c^
		Standard care	24/53 (45.2)		
	GLS	Enalapril	10/47 (21.2)	0.95 (0.33-2.74)	0.92^c^
		Standard care	9/41 (21.9)		
	LVEF	Enalapril	2/49 (4.1)	N/A	0.24^d^
		Standard care	0/48 (0.0)		
	Any cardiotoxicity per BSE/BCOS^e^	Enalapril	10/47 (21.2)	0.95 (0.33-2.74)	0.92^c^
		Standard care	9/41 (21.9)		
	Asymptomatic CTRCD cardiotoxicity per ESC^f^	Enalapril	42/49 (85.7)	0.55 (0.13-2.01)	0.37^c^
		Standard care	44/48 (91.6)		

Values are n/n (%), unless otherwise indicated.

BSE/BCOS = British Society of Echocardiography/British Cardio-Oncology Society; cTnI = cardiac troponin I; cTnT = cardiac troponin T; CTRCD = cancer therapy–related cardiac dysfunction; ESC = European Society of Cardiology; GLS = global longitudinal strain; LVEF = left ventricular ejection fraction; RD = risk difference; RR = risk ratio.

a Intention-to-treat analyses for all outcomes, adjusted for chemotherapy regimen.

b RD: −0.06 (95% CI: −0.20 to 0.08); RR: 0.89 (95% CI: 0.76-1.05).

c *P* value obtained from logistic regression.

d Obtained from Firth’s logistic regression.

e Includes all possible, probable, and definite cases of cardiotoxicity in accordance with the BSE/BCOS guidelines.

f Includes mild, moderate, and severe CTRCD calculated using cTnT.

**Figure 2:**
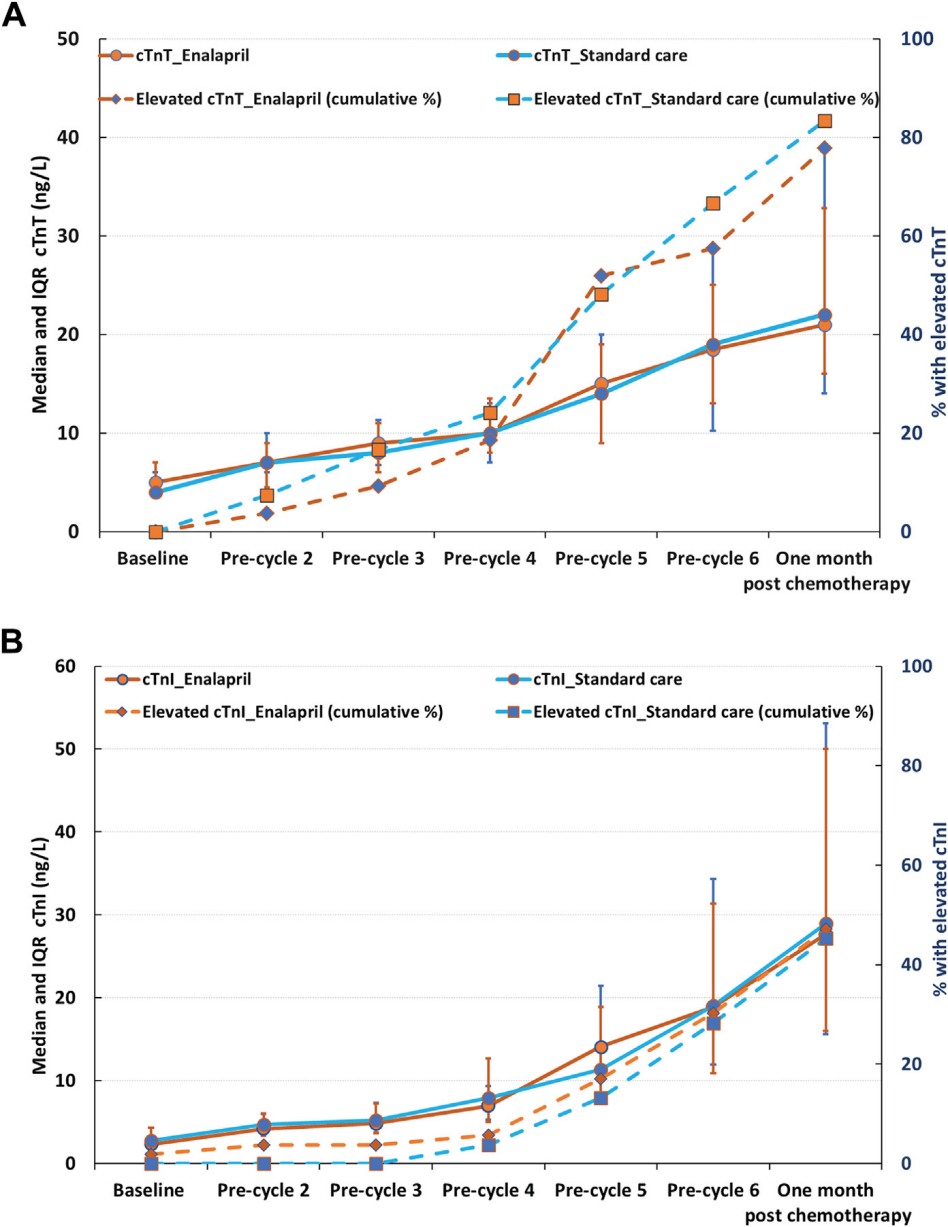
Median and Cumulative Incidence of Myocardial Injury by Chemotherapy Cycle This figure summarizes the changes in cTn across chemotherapy cycles, with samples were taken at baseline (before cycle 1), <72 hours before each subsequent cycle, and 1 month after the last dose of anthracycline. Solid lines represent median (Q1-Q3) troponin levels, and dashed lines show the cumulative percentage of patients developing myocardial injury (elevated cardiac troponin) at each timepoint. (A) Cardiac troponin T (cTnT) levels and (B) cardiac troponin I (cTnI) levels, both illustrating the increasing risk of myocardial injury with progressive anthracycline doses. Notably, there is no attenuation of this risk with enalapril treatment. Furthermore, the cumulative rates of myocardial injury differ between the cTnT and cTnI assays, markedly underscoring the variable sensitivity of these biomarkers.

cTnT concentrations consistently increased during anthracycline treatment without significant differences between the enalapril and standard care groups. One month after chemotherapy, the median cTnT concentration was 21 ng/L (Q1-Q3: 14 to 39 ng/L) in the enalapril group compared with 22 ng/L (Q1-Q3: 16 to 33 ng/L) in the standard care group (Figure 2A). No notable interactions were observed in the prespecified subgroup analyses, except within the EC90 chemotherapy regimen. Although given the small numbers, these findings should be interpreted with caution (Supplemental Figure 3).

Isolated troponin elevations were uncommon; after recording a value above the ULN, only 4 (3.7%) patients had a subsequent measurement below the ULN. Missing samples were uncommon, with 642 (91.5%) of 702 time points yielding successful cTnT measurements.

### Secondary Outcomes

#### Cardiac Troponin I

Myocardial injury, measured by cTnI levels during or 1 month after anthracycline chemotherapy, was comparable between the groups: 47.2% (n = 25 of 53) patients in the enalapril group and 45.3% (n = 24 of 53) in the standard care group (OR: 1.10; 95% CI: 0.50 to 2.38; *P* = 0.82) (Table 3). cTnI concentrations consistently increased during anthracycline treatment without significant differences between the groups. One month after chemotherapy, the median cTnI concentration was 28 ng/L (Q1-Q3: 16 to 53 ng/L) in the enalapril group and 29 ng/L (Q1-Q3: 16 to 50 ng/L) in the standard care group (Figure 2B).

#### Cardiac Function

**Figure 3:**
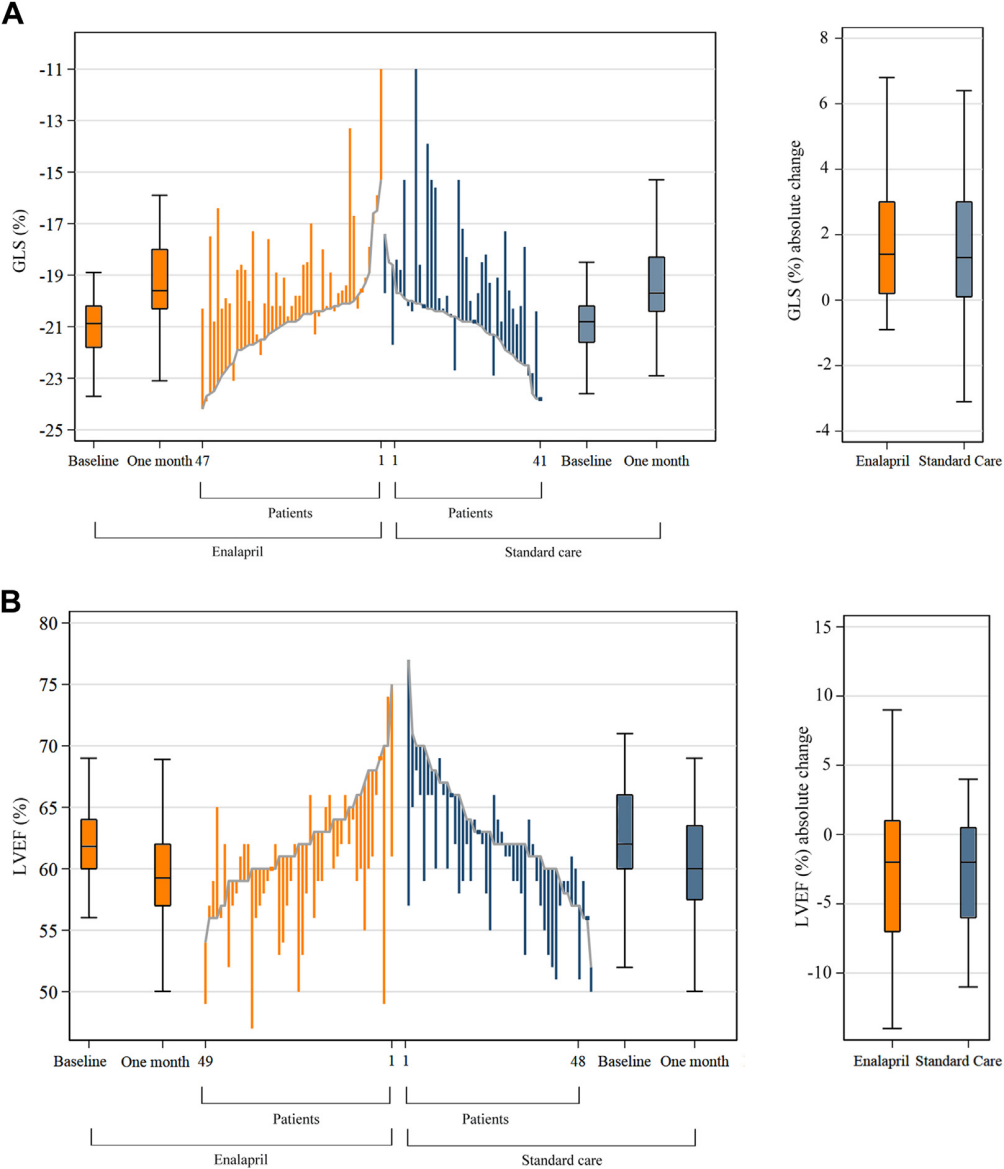
Changes by Group in Key Echocardiographic Parameters Over Time These line plots depict individual participant changes from baseline to 1 month after chemotherapy. (A) GLS changes, in which ascending lines indicate worsening LV function. (B) LVEF changes, in which descending lines indicate deterioration. For both panels, baseline values are arranged in ascending order for the enalapril group and descending for standard care. Boxplots show the first and third quartiles, with the median represented by a central solid line. Whiskers extend to the furthest points within 1.5 times the interquartile range from the quartiles. There were no significant differences in the absolute percentage changes in (A) GLS and (B) LVEF between the enalapril and standard care groups. Abbreviations as in Figure 1.

At baseline, GLS was similar in both groups, recorded at −20.8% (95% CI: −21.8% to −20.2%) for the enalapril group and −20.8% (95% CI: −21.6% to −20.3%) for the standard care group. A relative decline in GLS >15% from baseline occurred in 21.3% (n = 10 of 47) of the enalapril group and 21.9% (n = 9 of 41) of the standard care group (OR: 0.95; 95% CI: 0.33 to 2.74; *P* = 0.92). The median absolute change in GLS showed no significant difference between the groups: 1.4 (Q1-Q3: 0.2 to 3.0) for enalapril and 1.3 (Q1-Q3: 0.1 to 3.2) for standard care (Table 3, Figure 3A).

Baseline LVEF was consistent between groups, with the enalapril group at 62% (Q1-Q3: 60% to 64%) and the standard care group at 62% (Q1-Q3: 60% to 66%). The median absolute change in LVEF was −2% for both the enalapril group (Q1-Q3: −7% to 2%) and the standard care group (Q1-Q3: −6% to 1%), indicating no significant differences (Figure 3B). An absolute reduction in LVEF >10% to an absolute value <50%, occurred in 4.1% (n = 2 of 49) of the enalapril group and none (n = 0 of 48) in the standard care group (Figure 3B).

Cardiotoxicity rates, summarized in Table 3, show that 85.7% (n = 42 of 49) of patients in the enalapril group and 91.7% (n = 44 of 48) in the standard care group demonstrated asymptomatic CTRCD according to European Society of Cardiology (European Society of Cardiology) guidelines.^6^

#### Safety

A total of 60 serious adverse events were reported, equally distributed between the 2 groups (30 participants each). Only 3 events were possibly related to the trial intervention, with the majority of serious adverse events linked to cancer or recognized chemotherapy side effects (Supplemental Table 2). In the standard care group, 1 patient died due to progressive NHL. No heart failure events were reported during the study.

Nine patients in the enalapril group discontinued the medication before completing chemotherapy due to 2 cases of cough, 2 cases of symptomatic hypotension, 4 instances of poor tolerance to chemotherapy, and 1 case of angioedema that recurred with chemotherapy after stopping enalapril.

Adverse events were notably more frequent in the enalapril group (Supplemental Table 3), with 37 adverse reactions specifically reported as related to enalapril (Supplemental Table 4). Despite this, no major safety concerns associated with enalapril were observed during the trial.

## Discussion

The PROACT trial found that enalapril does not prevent CTRCD in patients treated with high-dose anthracycline for breast cancer or NHL. The Central Illustration summarizes consistent findings across myocardial injury and cardiac function outcomes. Notably, there was a clear dose-response relationship observed with increasing anthracycline cycles for both cTnT and cTnI, yet the response curves for enalapril and standard care overlapped, indicating no differential benefit from enalapril.

**Central Illustration:**
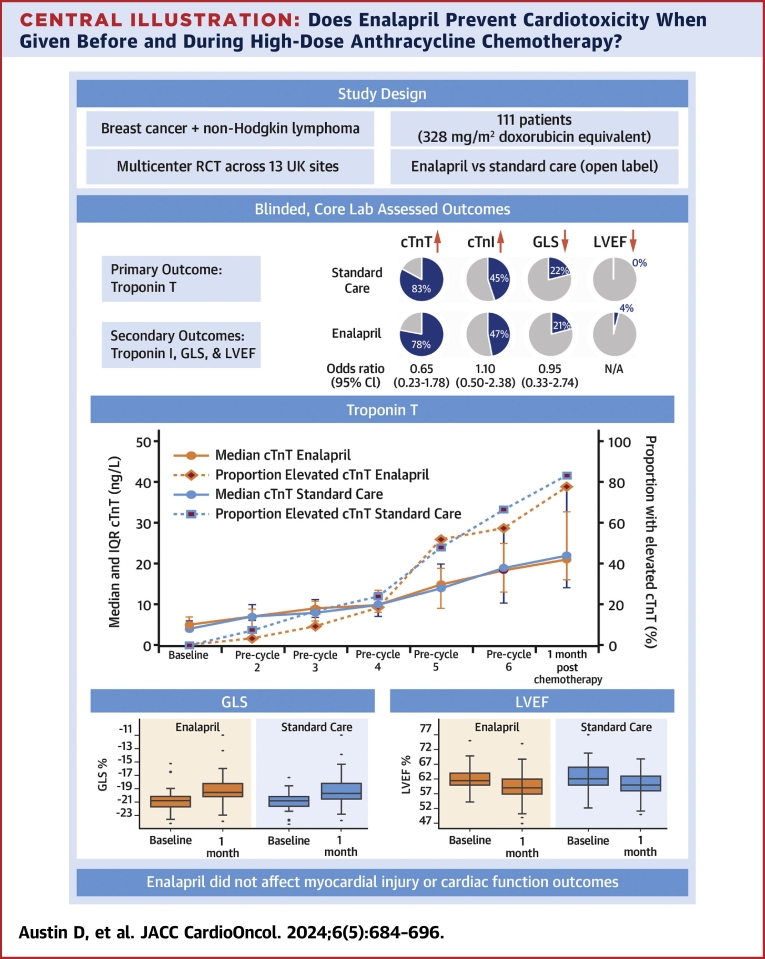
Does Enalapril Prevent Cardiotoxicity When Given Before and During High-Dose Anthracycline Chemotherapy? The PROACT (Preventing Cardiac Damage in Patients Treated for Breast Cancer and Lymphoma) trial did not find evidence supporting the use of enalapril to prevent cardiotoxicity in patients receiving high-dose anthracycline chemotherapy for breast cancer or non-Hodgkin lymphoma. cTnI = cardiac troponin I; cTnT = cardiac troponin T; GLS = left ventricular global longitudinal strain; LVEF = left ventricular ejection fraction; RCT = randomized controlled trial.

The PROACT trial exhibits several strengths that enhance its validity. First, it recruited an enriched population based on anthracycline dose, aimed at assessing those at increased risk of cardiotoxicity. Second, all cardiac biomarker and echocardiographic assessments were conducted by blinded core laboratories, ensuring unbiased and objective evaluations of enalapril’s effectiveness. Furthermore, the trial included a wide demographic by recruiting patients from multiple centers. Enalapril was titrated to a mean daily dose of 17.7 mg, aligning with doses used in pivotal heart failure studies, providing a robust basis to test its effectiveness in this context.^23^

Consistent with previous studies on RAAS inhibition, no major safety issues were observed with enalapril.^24^^,^^25^ However, discontinuations during chemotherapy occurred in 16% of the patients in the treatment group, with adverse events more frequently reported among those treated with enalapril. Notably, 4 of these 9 patients discontinued both enalapril and chemotherapy concurrently due to intolerance, similar to findings from the open-label Cardiac Care Trial study.^25^

Understanding cardiotoxicity rates is crucial for designing clinical trials with sufficient power in this field. Baseline cardiovascular risk and the administered dose of anthracycline significantly determine an individual’s risk.^26^ In modern oncology practice, particularly for breast cancer, there has been a shift toward lower anthracycline doses within combination therapy regimens.^27^ Consequently, recent cardio-oncology trials have typically included a broader range of participants, regardless of the anthracycline dose received. However, many of these trials involve participants receiving doses <250 mg/m^2^ doxorubicin equivalents. This tendency toward lower doses results in less cardiotoxicity, which in turn results in insufficient statistical power to discern any protective effects of interventions.^24^^,^^28^, ^29^, ^30^

In 2 previous multicenter randomized controlled trials assessing atorvastatin, the STOP CA Randomized Clinical Trial attributed its positive result to the higher rates of cardiotoxicity observed in their older population receiving higher anthracycline dosage (50 years and 264 mg/m^2^) compared with the study by Hundley et al.^31^^,^^32^ The PROACT trial included participants who were older still, with even higher received anthracycline doses (58 years and 328 mg/m^2^), and was therefore specifically designed to assess cardiotoxicity and the potential effects of enalapril in a higher risk population.^32^

Baseline clinical risk, calculated retrospectively using the Heart Failure Association/International Cardio-Oncology Society risk score, which was developed after the trial’s commencement, showed that 40% of the PROACT trial patients were at moderate risk and 12% at high risk of CTRCD.^26^ However, the very high risk category patients, often already indicated for RAAS inhibition due to pre-existing conditions, were not included in this study. Despite this, the equivalent rates of serious adverse events between the enalapril and control groups offer reassurance, suggesting that tailored approaches remain viable. Nonetheless, the high incidence of myocardial injury and early reductions in GLS observed confirm that the study population was at a significant risk of developing late cardiotoxicity.

The choice of primary and secondary endpoints was based on a contemporary understanding of anthracycline cardiotoxicity. Patients showing increased concentrations of cTn without significant changes in LVEF meet the European Society of Cardiology criteria for mild CTRCD.^6^ An important finding was the markedly lower myocardial injury rates identified using the cTnI assay compared with the cTnT assay at matched timepoints. Within the PROACT trial, 89% (n = 86 of 97) of patients met the criteria for mild CTRCD based on cTnT levels, whereas only 51% (n = 49 of 96) did so based on cTnI. This discrepancy raises concerns about the reliability of defining cardiotoxicity solely by cTn upper reference limits in routine clinical practice, in which centers usually employ just 1 type of assay. Mecinaj et al^33^ suggest potential explanations for the observed disparities between cTnT and cTnI, including variations in release kinetics and differences in the biological equivalence of the 99th percentile across assays and platforms. Further research is essential to understand these discrepancies and to establish clinically significant thresholds for the various high-sensitivity assays available.

The PROACT trial contributes to the body of knowledge on potential treatments aimed at reducing the cardiovascular impact of anthracycline therapy. Previous meta-analyses have suggested a small potential benefit of various neurohormonal therapies across a range of cancer chemotherapy regimens. However, these analyses also highlighted that most studies were single-center studies, with a high risk of bias.^34^ Notably, a previous study showed rapid normalization of troponin levels and subsequent prevention of LV decline with enalapril, results that the PROACT trial did not replicate. This discrepancy may stem from differences in treatment strategy (pretreatment vs troponin triggered) and the duration of enalapril therapy.^10^ Additionally, a more recent multicenter study found no advantage of a troponin-triggered strategy over standard care.^25^ Although clinical endpoint studies, such as the new diagnosis of heart failure or LVEF <40%, have been proposed, they would require considerably larger sample sizes than those used to date.^35^ Given the PROACT trial’s findings, a clinical endpoint study using enalapril as a preventative intervention during chemotherapy would not be justified.

### Study Limitations

The PROACT trial was open label and not placebo controlled, which may introduce bias. To mitigate this, primary and key secondary endpoints were independently assessed by core laboratories that were blinded to participant allocation. However, the open label design may have influenced adverse events, potentially contributing to a nocebo effect. This effect might be particularly pronounced among the small subset of patients who were not tolerating chemotherapy, along with the observed higher number of adverse events in the enalapril group.^24^^,^^25^

Second, the trial’s statistical power was reduced from 90% to 80% due to complex recruitment challenges. The increased use of tumor profiling and the introduction of alternative treatment regimens for HER2-positive patients, along with updated UK guidelines favoring lower-dose anthracycline regimens, reduced the number of eligible breast cancer patients.^27^^,^^36^^,^^37^ Consequently, the inclusion of patients with NHL became necessary, many of whom required more urgent treatment or presented with myocardial injury at baseline. Furthermore, recruitment was severely affected by the COVID-19 pandemic, which required a pause to recruitment. Reducing the statistical power to 80% allowed for a smaller required sample size, with a minimum of 106 patients needed to provide data for the study’s endpoint assessments, a target that was ultimately reached. Despite the recruitment challenges and a very low attrition rate within the trial (<5%), the number of primary endpoint events was higher than expected. This suggests that the main findings of the study are likely unaffected by the open-label design or the reduced sample size. This assessment is supported by sensitivity analyses and consistent findings across secondary endpoints.

Third, the trial included participants with 2 types of cancer. Subgroup analyses by cancer type did not change the interpretation of the primary outcomes, affirming the relevance of anthracycline cardiotoxicity even beyond the populations studied in the PROACT trial. Finally, the assessment of echocardiographic endpoints was conducted at an early postchemotherapy stage, and longer-term follow-up is needed.

## Conclusions

Adding enalapril to standard care did not demonstrate superiority in preventing cardiotoxicity among patients receiving high-dose anthracycline-based chemotherapy. Thus, the PROACT trial does not support the use of enalapril in this setting.

Perspectives **COMPETENCY IN MEDICAL KNOWLEDGE OR PATIENT CARE:** The PROACT randomized controlled trial, which included 111 patients receiving ≥300 mg/m^2^ doxorubicin equivalent chemotherapy for breast cancer or NHL, demonstrated that enalapril, administered at a mean dose of 17.7 mg daily, did not protect against markers of cardiac injury such as cTns or cardiac dysfunction.

**TRANSLATIONAL OUTLOOK:** The findings from the PROACT randomized controlled trial indicate that further clinical endpoint trials investigating enalapril as preventative treatment during anthracycline chemotherapy would not be justified. Alternative cardioprotective strategies should be explored to prevent long-term heart failure morbidity and mortality in this vulnerable patient group.

## Funding Support and Author Disclosures

This work was supported by the National Institute for Health and Care Research (PB-PG-0815-20061). Dr Gilbert was supported by a grant from JGW Patterson Foundation. Dr Mills was supported by a Chair Award (CH/F/21/90010), Programme Grant (RG/20/10/34966), and Research Excellence Award (RE/24/130012) from the British Heart Foundation. Dr Austin has received speaker fees from Philips Volcano, AstraZeneca, and Pfizer; and research grants awarded to Newcastle University from TA Sciences, Kancera, and AstraZeneca. Dr Maier has received research grants awarded to Newcastle University from TA Sciences, Kancera, and AstraZeneca. Dr Maddox has received funding to attend meetings from Novartis and AbbVie. Dr Mills has received research grants awarded to the University of Edinburgh from Abbott Diagnostics, Siemens Healthineers, and Roche Diagnostics, outside the submitted work; and honoraria from Abbott Diagnostics, Siemens Healthineers, Roche Diagnostics, LumiraDx, and Psyros Diagnostics. Dr Kasim was an employee of Durham University during his involvement in the PROACT trial, and is now an employee of GlaxoSmithKline. Dr Plummer has received speaker fees or travel expenses from Amgen, BeiGene, Calgene, Incyte, Ipsen, Novartis, and Servier. All other authors have reported that they have no relationships relevant to the contents of this paper to disclose.
