# Supplementary material for: Preventing Cardiac Damage in Patients Treated for Breast Cancer and Lymphoma: The PROACT Clinical Trial
Source: JACC CardioOncol. 2024 Aug 27;6(5):684–96. doi: 10.1016/j.jaccao.2024.07.010 (PMC11520224; doi:10.1016/j.jaccao.2024.07.010)
Supplement: Supplemental Material [file mmc1.docx]

**Preventing Cardiac Damage In Patients Treated For Breast Cancer And Lymphoma: A Phase 3 Randomized, Open Label, Blinded Endpoint, Superiority Trial Of Enalapril To Prevent Anthracycline-induced Cardiotoxicity (PROACT)**

David Austin MB ChB, MD^1,2^; Rebecca H Maier MSc^1,2^; Nasima Akhter PhD^3^; Mohammad Sayari MSc^4^; Emmanuel Ogundimu PhD^4^; Jamie M Maddox MB ChB^5^; Sharareh Vahabi MB ChB, MD^6^; Alison C Humphreys MB BcH, MD^7^; Janine Graham MB ChB^7,8^; Helen Oxenham MB ChB, MD^9^; Sophie Haney MBBS^7,10^; Nicola Cresti MD, PhD^11^; Mark Verrill MB, BChir^11^; Wendy Osborne MBBS^11^; Kathryn L Wright MB ChB^11,12^; Rebecca Goranova MBBS^13^; James R Bailey MBBS PhD^14^; Nagesh Kalakonda MBBS, PhD^15^; Mac Macheta MB ChB^16^; Mari F Kilner MB ChB^17^; Moya E Young MBBS^18^; Nick J Morley MBBS^19^; Pratap Neelakantan MBBS, MD^20^; Georgia Gilbert MB BS^1^; Byju K Thomas BSc^1^; Richard J Graham MBChB^1^; Takeshi Fujisawa PhD^21^; Nicholas L Mills MB ChB, PhD^21^; Victoria Hildreth PhD^22^; Jonathan Prichard MSc^22^; Adetayo S Kasim PhD^23^; Helen C Hancock PhD^22^; Chris Plummer BM, BCh, PhD^24,25^

1. Academic Cardiovascular Unit, The James Cook University Hospital, South Tees Hospitals NHS Foundation Trust, Middlesbrough, UK 2. Population Health Sciences Institute, Newcastle University, Newcastle-upon-Tyne, UK 3. School of Health and Life Sciences, Teesside University, Middlesbrough, United Kingdom, UK 4. Department of Mathematical Science, Durham University, Durham, UK 5. Department of Haematology, South Tees Hospitals NHS Foundation Trust, Middlesbrough, UK 6. Bristol Heart Institute, University Hospitals Bristol and Weston NHS Foundation Trust, Bristol, UK 7. Cancer Services, South Tees Hospitals NHS Foundation Trust, Middlesbrough, UK 8. Cancer Services, North Tees and Hartlepool NHS Foundation Trust, Stockton-on-Tees, UK 9. Department of Cardiology, North Tees and Hartlepool NHS Foundation Trust, Stockton-on-Tees, UK 10. Cancer Services, County Durham and Darlington NHS Foundation Trust, UK 11. Freeman Hospital, Newcastle upon Tyne Hospitals NHS Foundation Trust, Newcastle upon Tyne, UK 12. Cancer Services, South Tyneside and Sunderland NHS Foundation Trust, Sunderland, UK 13. Department of Oncology, Derriford Hospital, University Hospitals Plymouth NHS Trust, Plymouth, UK 14. Department of Haematology, Hull University Teaching Hospitals NHS Trust, The Queen’s Centre for Oncology & Haematology, Castle Hill Hospital, Hull, UK 15. Department of Molecular and Clinical Cancer Medicine, University of Liverpool, Liverpool UK 16. Department of Haematology, Blackpool Teaching Hospitals, Blackpool, UK 17. Pathology Services, Northumbria Healthcare NHS Foundation Trust, North Shields, UK 18. Department of Haematology, East Kent Hospitals University NHS Foundation Trust, Canterbury, UK 19. Department of Haematology, Sheffield Teaching Hospitals NHS Foundation Trust, Sheffield, UK 20. Department of Haematology, Royal Berkshire NHS Foundation Trust, Reading, UK 21. [Centre for Cardiovascular Science](https://www.research.ed.ac.uk/en/organisations/centre-for-cardiovascular-science), University of Edinburgh, Edinburgh, UK 22. Newcastle Clinical Trials Unit, Newcastle University, Newcastle upon Tyne, UK. 23. Department of Anthropology, Durham University, Durham, UK 24. Department of Cardiology, The Newcastle upon Tyne Hospitals NHS Foundation Trust, Newcastle upon Tyne, UK. 25. Faculty of Medical Sciences, Newcastle University, Newcastle upon Tyne, UK.

**Corresponding Author:**

Dr David Austin, BSc MedSci MD FRCP (Glasg), Academic Cardiovascular Unit, The James Cook University Hospital, South Tees Hospitals NHS Foundation Trust, Marton Road, Middlesbrough, United Kingdom, TS4 3BW ([david.austin@nhs.net](mailto:david.austin@nhs.net))

Table of Contents

[Statistical Analysis Plan - Abridged 4](#_Toc174864035)

[Trial committees 11](#_Toc174864036)

[Recruiting Centres 12](#_Toc174864037)

[Core Laboratories 13](#_Toc174864038)

[Collaborators 13](#_Toc174864039)

[Full Inclusion and Exclusion Criteria 14](#_Toc174864040)

[Table 1 Baseline Cancer Sub-types 17](#_Toc174864041)

[Table 2 Serious adverse events 18](#_Toc174864042)

[Table 3 Frequency and grade of all adverse events 20](#_Toc174864043)

[Table 4 Adverse events (adverse reactions) considered to be related to enalapril 21](#_Toc174864044)

[Figure 1 Mean Blood Pressure (95% CI) by randomisation group 22](#_Toc174864045)

[Figure 2 Intention-To-Treat, Per-Protocol and As-Treated analyses of the primary outcome 22](#_Toc174864046)

[Figure 3. Forest plot of regression results for primary outcome by intervention group and other baseline characteristics 24](#_Toc174864047)

# Statistical Analysis Plan - Abridged

| TRIAL FULL TITLE | Preventing cardiac damage in patients treated for breast cancer and lymphoma: a phase 3 Randomised, Open label, blinded endpoint, superiority trial of enalapril to prevent Anthracycline-induced CardioToxicity (PROACT). |
| --- | --- |
| Study design | A multi-centre, prospective, randomised, open-label, blinded end-point, superiority trial |
| EUDRACT NUMBER | 2017-001094-16 |
| ClinicalTrials.gov | NCT03265574 |
| SAP VERSION | Extracted from the final SAP, version 1.0, dated 10/03/2023 |
| TRIAL STATISTICIAN | Dr Nasima Akhter |
| TRIAL CHIEF INVESTIGATOR | Dr David Austin |
| SAP AUTHORS | Dr Nasima Akhter, Professor Adetayo Kasim,  Dr Emmauel Ogundimu |

1. **General Study Design and Plan**

**Summary:** The study is a prospective, randomised, open-label, blinded end-point, superiority trial in patients undergoing anthracycline-based chemotherapy for breast cancer or NHL. Patients were randomised in a 1:1 ratio to enalapril (intervention) or control (standard care). The trial will evaluate the effectiveness of enalapril in preventing cardiotoxicity detected by cardiac troponin T.

The trial will randomise 106 patients (plus attrition) due to receive anthracycline-based chemotherapy for breast cancer or NHL at participating NHS Trusts.

1. **PURPOSE OF THE ANALYSES**

These analyses will assess the effectiveness of enalapril in preventing cardiotoxicity in patients receiving anthracycline-based chemotherapy for breast cancer or NHL, in comparison with standard care (no enalapril).

1. **RANDOMISATION AND BLINDING**

1. **Randomisation**

Randomisation is performed using a minimisation scheme to ensure patients randomised to each group are comparable at baseline. The minimisation scheme will account for the planned 6 cycle chemotherapy regimen (Epirubicin and Cyclophosphamide (EC 90) or Fluorouracil, Epirubicin and Cyclophosphamide (FEC 75)), or R-CHOP or CHOP (Cyclophosphamide, Doxorubicin, Vincritsine and Prednisolone +/- Rituximab), and, in breast cancer, HER2 (human epidermal growth factor receptor 2) status (positive or negative).

Patients who will receive an alternative anti-CD20 monoclonal antibody (for example O-CHOP) or a combination of rituximab with polatuzumab (e.g polatuzumab-R-CHP)), are eligible as long as the total planned doxorubicin dose is ≥300mg/m^2^ over 6 cycles.

Eligible patients will be randomised by delegated and trained members of the research team at each centre using a 24-hour, central, secure, web-based randomisation system with concealed allocation. Eligible patients will be randomised in a 1:1 ratio to receive standard care plus enalapril maleate (intervention under study) or standard care (control).

1. **Blinding**

The trial employs a prospective randomised blinded endpoint design. Analysis of troponin T as the primary outcome measure, of the echocardiogram and secondary outcome measure troponin I, will be completed by staff who are blinded to the patients’ trial allocation, as detailed in the trial PROACT laboratory manual. Central laboratories in Newcastle upon Tyne will analyse Troponin T. Troponin I will be analysed separately in suitable facilities. All echocardiograms will be sent securely to the Echo Core laboratory, based at The James Cook University Hospital for review and reporting.

1. **Sample Size**

1. **SAMPLE SIZE ESTIMATION**

Pilot data from 36 patients with breast cancer and 143 samples, showed a positive troponin T in 47% of patients who received >300mg/m^2^ of epirubicin in a six-cycle regimen. As a sensitive marker of cardiac cell death, we expect troponin release to be “turned off” if enalapril is effective. There are other potential causes of an elevated troponin during chemotherapy that are accounted for in the sample size calculation. There is clinical consensus that a rate of 20% in elevated troponin would fully account for other causes such as infection or disease progression.

The initial recruitment target was 170 participants, based on a sample size estimation for the study assuming a level of significance (alpha) of 5%, and 90% power indicating that 140 patients are needed to detect a reduction in the proportion of patients with cardiac troponin T present from 47% to 20% using a two-sided Fisher’s exact test. Allowing for 20% attrition, the trial planned to recruit and randomise 170 patients. There is consensus within the clinical community that, a large effect size will be necessary to convince the clinical community to change the pathway of care for these patients.

1. **SAMPLE SIZE RE-ESTIMATION AND EXTENSION**

Due to reduced recruitment rates, and in agreement with the funder and sponsor, the sample size has been reduced to 106 participants, plus attrition. The sample size re-calculation was performed by the trial statistician using the same parameters as for the original sample size (two-sided Fisher’s exact test), but with a reduced power of 80%. The statistician had no access to trial data for the sample size re-estimation. A re-estimation of sample size at reduced power of 80%, which assumed a level of significance (alpha level) of 5%, to detect a difference between 47% and 20%, indicated that PROACT will require 106 patients, plus attrition. The estimation was done using G*Power version 3.1.

The trial aims to recruit and randomise a sample of 106 patients adjusted for additional patients to account for attrition, who will receive anthracycline-based chemotherapy for their breast cancer or NHL at participating NHS Trusts. It is anticipated that a minimum sample of 110-112 will be adequate to retain 80% power with current rate of attrition (4-6%).

1. **General Considerations**

The analysis for the main trial will be performed at the end of the trial (at primary endpoint), and after the Statistical Analysis Plan (SAP) has been approved by the Chief Investigator and reviewed by the Data Monitoring Committee. No interim analysis will be conducted. Some of the exploratory analyses that are pre-specified may be conducted at a later date.

1. **ANALYSIS POPULATIONS**

The main analysis of primary and secondary outcomes will follow intention to treat principles with patient data analysed according to randomisation and irrespective of intervention received. Analysis of some secondary outcomes will be undertaken as change from baseline. Other analysis groups, such as per-protocol, may be considered subsequently. Follow-up data at one year will be analysed and reported separately.

All available cases will be included in the intention to treat analyses, irrespective of when a patient may have stopped chemotherapy and/or enalapril at different cycles. Attrition will be defined as patients not contributing to the primary outcome (post-baseline data not available for cTnT). The primary analysis will include all cTnT samples taken and analysed in the laboratory. A minimum of 106 patients analysed would retain 80% power for the study.

Data from patients who stopped chemotherapy, stopped enalapril, or withdrew, will remain in the overall analysis. Unless the patient withdraws consent, further data may continue to be collected from their medical records to ensure complete trial data, and samples collected for future research may still be analysed.

1. **Full Analysis Population**

Following the intention to treat principle, all patients who consented to the trial and who were randomised, will be included in the analyses.

All those randomised to the intervention and control groups and who have contributed data will be included in the main analysis, including those with protocol deviations or violations.

1. **Per protocol and as treated population**

Separate analysis populations of “as treated” and “per protocol” will be undertaken as a sensitivity analysis. The protocol violation regarding samples collected in EDTA tubes at one centre, will be included as analysed in the primary analysis. For the per protocol analysis, these patients will be excluded. The per protocol group will be defined as participants who have received a minimum of 4 chemotherapy cycles and must include at least one of cycles 5 or 6 or EOS visit. For participants on the enalapril arm, they must not have withdrawn from enalapril.

1. **Safety Population**

Apart from those who withdrew prior to receiving any treatment, all patients who received treatment in either arm, enalapril and standard care, will be assessed for safety.

1. **INTERIM ANALYSIS**

No interim analysis of data is planned.

1. **MULTIPLE TESTING**

Analysis of the primary outcome will reflect key findings of the trial and analysis of secondary outcomes will provide supplementary data only. Therefore, no multiplicity corrections for multiple testing will be applied.

1. **STATISTICAL SOFTWARE**

All analyses will be done using R studio and SPSS version 27.0.

1. **Trial Outcomes**
2. **PRIMARY OUTCOME**

The primary outcome is the presence (≥14ng/L) or absence (<14/L) of cTnT release at any time during anthracycline treatment, and at one month after the last dose of anthracycline (cycles 2-6, end of study); the cut-off value is based on the Roche assay (Giannitsis E,  2010, Sandoval et al., 2022).

1. **Computation of primary outcome:**

A binary variable will be created using data from all cycles (2,3,4,5,6) and end of study to identify whether the patient had the presence (≥14ng/L) or absence (<14/L) of cardiac TnT release at any time. As per the 2021 AHA/ACC chest pain guidelines (Giannitsis E et al 2010, Sandoval et al., 2022,), if cardiac troponin T release during these time points is ≥14ng/L (section 7.1.1), cardiac TnT release will be coded as 1, otherwise it will be coded as 0 (absence of increased cardiac troponin T at any time).

1. **Primary Effectiveness Analysis**

The null hypothesis is that there will be no difference in cardiotoxicity measured using cTnT release between the intervention (patients with breast cancer and NHL treated with anthracycline-based chemotherapy and enalapril) and control group (those receiving anthracycline-based chemotherapy only). The primary analysis of assessment of the presence or absence of cTnT release will apply the intention-to-treat principle on the full population. The analysis will use a logistic regression, accounting for minimisation factors that are available across all participant types (breast cancer and NHL patients).

The cluster effect will be ignored since site level variability is not expected due to chemotherapy being standard across sites, and cTnT being analysed centrally, meaning no site level variability in cTnT is expected. Furthermore, Kahan and Morris (2013) suggested that cluster effects can be ignored if they were not used in the randomisation and therefore type I error rates would be correct irrespective of whether the cluster is accounted for in the analysis. They argued that for situations when a centre was not balanced in the randomisation in a multicentre trial, an analysis ignoring centre effects will produce unbiased standard errors and correct type I error rates (Kahan & Morris, 2013).

Study groups will be tested at the 2-sided significance level. Analysis will be undertaken on the full analysis population. The odds ratio and confidence intervals will be reported for the binary outcome. All results, including p-values will be reported to 2 decimal places.

1. **SECONDARY OUTCOMES:**

1. **Cardiac Troponin I (cTnI)**

The secondary cTnI outcome is defined as the presence (>26.2ng/L) or absence (≤26.2ng/L) of cTnI release at any time during anthracycline treatment, and at one month after the last dose of anthracycline (cycles 2-6, end of study)(Welsh et al., 2019); the cut-off value is based on the Abbott ARCHITECT STAT assay (NICE, 2020).

1. ***Computation of cTnI secondary outcome:***

A binary variable will be created using data from all cycles (2,3,4,5,6) and end of study to identify whether the patient had presence (>26.2ng/L) or absence (≤26.2ng/L) of cardiac TnI release at any time. If cardiac troponin I release during these time points is >26.2ng/L, cTnI release will be coded as 1, otherwise it will be coded as 0 (absence of increased cardiac troponin I at any time).

1. **Echocardiographic outcomes**

Analysis between the intervention (patients with anthracycline-based chemotherapy and enalapril,) and control group (those receiving anthracycline-based chemotherapy alone) for the following derived echocardiographic secondary endpoints:

1. % change in GLS from baseline to end of study (continuous end point)
2. % change in LVEF from baseline to end of study (continuous end point)
3. a relative decline of >15% (Dobson et al., 2021) in GLS from baseline to end of study (Dobson et al., 2021; Plana et al., 2014)(dichotomised endpoint) (appendix 4)
4. a decline of >10 absolute percentage points from baseline LVEF to a value of <50%* (appendix 3)(Cardinale et al., 2015; Cardinale et al., 2020; Dobson et al., 2021) (dichotomised endpoint)*
5. yes to either (c) or (d)**

*synonymous with definite cardiotoxicity

** synonymous with definite or probable cardiotoxicity

Specific analysis of the echocardiographic endpoints will be dependent upon the nature of the specific endpoint and data structure. For example, c, d and e are binary outcomes reflecting abnormal change from baseline using a logistic regression model adjusted for minimisation factors. Descriptive results will show average values of GLS and LVEF over time by plotting median and interquartile range by data collection points, as box plots (median and interquartile range) or error bars (mean and confidence intervals). Changes over time (one month after baseline values) will also be shown as descriptive result. To assess whether secondary outcome continuous endpoints: a) % change in GLS from baseline to end of study (continuous endpoint),  and LVEF: b) % change in LVEF from baseline to end of study (continuous endpoint), varied significantly by groups, a linear mixed effect model using those continuous outcomes adjusting for minimisation factors will be used. Some of the results will be presented as figures. Echo data (pre and post EF, and GLS) will be presented as a figure reflecting change from baseline to end of study, as demonstrated by Fig 2 SUCCOUR study (Thavendiranathan et al., 2021).

1. **Descriptive and exploratory Analyses**

Continuous data will be summarised by study group using the following statistics: n, mean±SD, median±IQR (for skewed data), minimum and maximum; whereas frequencies and percentages (based on non-missing sample size) will be used to summarise categorical data. Overall, all data will be listed with separate tables for baseline and demographic variables, key variables and safety assessment tables. Descriptive analyses will include tolerance of enalapril, median dose of enalapril, and tabulation of AR and AEs. All summary tables will be structured with a column for each study group and will be annotated with the total population size relevant to that table/treatment, including any missing observations. Cancer and chemotherapy details, such as whether patients completed the planned course of chemotherapy, had dose reductions, dose delays, reasons for reduction or delays, and any reported disease progression, will also be tabulated. Results will also be presented in figures, as appropriate.

We will tabulate baseline cardiotoxicity risk overall, and by group, as per the ESC guidelines (Table 4, Lyon 2022, ESC guidelines on cardio-oncology). Cardiotoxicity equivalent dose will be calculated as per table 5 in ESC guidelines on cardio-oncology and tabulated as epirubicin and doxorubicin equivalent overall.

1. **SENSITIVITY ANALYSIS**

**As treated**: The as-treated analysis will be considered for sensitivity analysis. All patients will be analysed based only on the actual intervention they received instead of treatment groups they were randomised to. A similar model as that used for the primary outcome effectiveness analysis will be repeated on this population.

**Per-Protocol:** The per-protocol analysis will include all subjects who adhered to the inclusion and exclusion criteria in the protocol and completed the main study (4-week follow up). This will mean that all subjects who did not deviate from the protocol will be included here. The per protocol group will be defined as participants who have received a minimum of 4 chemotherapy cycles and must include cycles 5 & 6 and/or EOS visit. For participants on the enalapril arm, they must not have withdrawn from enalapril. The per-protocol population will usually be smaller than the full analysis population due to the removal of data for those patients who violated the trial protocol according to the above definition for this per-protocol analysis. Similar model used for the primary outcome effectiveness analysis will be repeated on this population.

An additional sensitivity analysis with a correction factor (based on difference from available samples) applied to them, will be undertaken. The analysis will apply a correction factor for the three cases where EDTA samples were taken in error.  This will include analysis of the complete data, including cases where EDTA samples were used in error. In those cases, EDTA based values would be imputed using their SST tube values and applying correction factor from available data where we have both EDTA and SST data.

The primary outcome will be analysed using generalised estimating equation (GEE) with binomial distribution and logit link, for the sensitivity analysis. Sensitivity analysis for primary outcomes will be performed using GEE as a population average model instead of mixed effect logistic regression, because of ease of interpretability.

Sensitivity analysis will also be performed to:

a) assess the effect of adherence to the intervention (enalapril) on the primary outcome,

b) compare outcomes in the patients with breast cancer and NHL

c) Assess impact on the primary outcome for those who received 4 cycles of chemotherapy or more.

1. **COVARIATES AND SUBGROUPS ANALYSIS**

The variables used in the randomisation (minimisation) scheme will be included in all analyses to adjust for accidental bias.

Subgroup analyses will be conducted according to

- Breast or lymphoma
- chemo regimen: EC 90; FEC 75 or CHOP
- Baseline EF: 50-55%, >55%
- Age above and below median, Age above and below 65
- Smoker: yes, no
- Chest Radiation therapy: yes/ no; and left/ right/ no.
- Hypertension: yes/no
- Diabetes: yes/no
- Known coronary heart disease: yes/no
- Known PVD: yes/no
- Any baseline cardiac risk: (yes to any known coronary heart disease, PVD, previous TIA/CVA, smoker, hypertension or diabetes and age >65)

1. **Summary of Demographic and Baseline Data**

Descriptive statistics will be used to summarise all demographic and baseline data. In general, all data will be listed with separate tables for the demographic variables, baseline variables and safety assessment tables.  All summary tables will be structured with a column for each study group (enalapril and standard care) and will be annotated with the total population size relevant to that table/treatment, including any missing observations. Univariable summary statistics and regression models will use available cases. Depending on the level of missingness for the primary outcome, sensitivity analysis for the impact of missing data will be performed on the primary outcome by using multiple imputation techniques. Descriptive analysis will include tolerance of enalapril, and tabulation of adverse reactions.

1. **References**

Cardinale, D., Colombo, A., Bacchiani, G., Tedeschi, I., Meroni, C. A., Veglia, F., Civelli, M., Lamantia, G., Colombo, N., Curigliano, G., Fiorentini, C., & Cipolla, C. M. (2015). Early detection of anthracycline cardiotoxicity and improvement with heart failure therapy. Circulation, 131(22), 1981-1988. https://doi.org/10.1161/circulationaha.114.013777

Cardinale, D., Iacopo, F., & Cipolla, C. M. (2020). Cardiotoxicity of anthracyclines. Frontiers in cardiovascular medicine, 7, 26.

Dobson, R., Ghosh, A. K., Ky, B., Marwick, T., Stout, M., Harkness, A., Steeds, R., Robinson, S., Oxborough, D., & Adlam, D. (2021). BSE and BCOS guideline for transthoracic echocardiographic assessment of adult cancer patients receiving anthracyclines and/or trastuzumab. Cardio Oncology, 3(1), 1-16.

Fournier, S., Iten, L., Marques-Vidal, P., Boulat, O., Bardy, D., Beggah, A., Calderara, R., Morawiec, B., Lauriers, N., & Monney, P. (2017). Circadian rhythm of blood cardiac troponin T concentration. Clinical Research in Cardiology, 106(12), 1026-1032.

Giannitsis E, Kurz K, Hallermayer K, Jarausch J, Jaffe AS, Katus HA. Analytical validation of a high-sensitivity cardiac troponin T assay. Clin Chem. 2010 Feb;56(2):254-61. doi:10.1373/clinchem.2009.132654.

Kahan, B. C., & Morris, T. P. (2013). Assessing potential sources of clustering in individually randomised trials. BMC medical research methodology, 13(1), 1-9.

Mueller-Hennessen, M., Lindahl, B., Giannitsis, E., Biener, M., Vafaie, M., deFilippi, C. R., Christ, M., Santalo-Bel, M., Panteghini, M., Plebani, M., Verschuren, F., Jernberg, T., French, J. K., Christenson, R. H., Body, R., McCord, J., Dilba, P., Katus, H. A., & Mueller, C. (2016). Diagnostic and prognostic implications using age- and gender-specific cut-offs for high-sensitivity cardiac troponin T — Sub-analysis from the TRAPID-AMI study. International Journal of Cardiology, 209, 26-33. https://doi.org/https://doi.org/10.1016/j.ijcard.2016.01.213

NICE. (2020). High-sensitivity troponin tests for the early rule out of NSTEMI. Diagnostics guidance. In.

Plana, J. C., Galderisi, M., Barac, A., Ewer, M. S., Ky, B., Scherrer-Crosbie, M., Ganame, J., Sebag, I. A., Agler, D. A., Badano, L. P., Banchs, J., Cardinale, D., Carver, J., Cerqueira, M., DeCara, J. M., Edvardsen, T., Flamm, S. D., Force, T., Griffin, B. P., . . . Lancellotti, P. (2014). Expert consensus for multimodality imaging evaluation of adult patients during and after cancer therapy: a report from the American Society of Echocardiography and the European Association of Cardiovascular Imaging. J Am Soc Echocardiogr, 27(9), 911-939. https://doi.org/10.1016/j.echo.2014.07.012

Sandoval, Y., Apple, F. S., Mahler, S. A., Body, R., Collinson, P. O., & Jaffe, A. S. (2022). High-Sensitivity Cardiac Troponin and the 2021 AHA/ACC/ASE/CHEST/SAEM/SCCT/SCMR Guidelines for the Evaluation and Diagnosis of Acute Chest Pain. Circulation, 146(7), 569-581. https://doi.org/doi:10.1161/CIRCULATIONAHA.122.059678

Thavendiranathan, P., Negishi, T., Somerset, E., Negishi, K., Penicka, M., Lemieux, J., Aakhus, S., Miyazaki, S., Shirazi, M., Galderisi, M., Marwick, T. H., Negishi, K., Costello, B., Wright, L., La Gerche, A., Mottram, P., Thomas, L., Shirazi, M., Penicka, M., . . . Kutty, S. (2021). Strain-Guided Management of Potentially Cardiotoxic Cancer Therapy. Journal of the American College of Cardiology, 77(4), 392-401. https://doi.org/https://doi.org/10.1016/j.jacc.2020.11.020

Thygesen, K., Mair, J., Giannitsis, E., Mueller, C., Lindahl, B., Blankenberg, S., Huber, K., Plebani, M., Biasucci, L. M., Tubaro, M., Collinson, P., Venge, P., Hasin, Y., Galvani, M., Koenig, W., Hamm, C., Alpert, J. S., Katus, H., Jaffe, A. S., & Care, t. S. G. o. B. i. C. o. t. E. W. G. o. A. C. (2012). How to use high-sensitivity cardiac troponins in acute cardiac care†. European Heart Journal, 33(18), 2252-2257. https://doi.org/10.1093/eurheartj/ehs154

Welsh, P., Preiss, D., Hayward, C., Shah, A. S. V., McAllister, D., Briggs, A., Boachie, C., McConnachie, A., Padmanabhan, S., Welsh, C., Woodward, M., Campbell, A., Porteous, D., Mills, N. L., & Sattar, N. (2019). Cardiac Troponin T and Troponin I in the General Population. Circulation, 139(24), 2754-2764. https://doi.org/10.1161/circulationaha.118.038529

# Trial committees

**Independent Data Monitoring and Ethics Committee**

Dr Alexander Lyon^1,2^ (Chair), Clinical Senior Lecturer and Honorary Consultant Cardiologist

Dr Anne Rigg^1^, Consultant Oncologist

Professor Alexander McConnachie^3^, Professor of Clinical Trial Biostatistics

**Independent Trial Steering Committee**

Dr Colette Jackson^4^ (Current Chair), Consultant Cardiologist

Dr Pam McKay^4^, Consultant Haematologist

Professor Helena Earl^5^ (former Chair*), Emeritus Professor of Clinical Cancer Medicine

Sarah DaCosta Evans^6^, Patient Representative

David Iles^6^, Patient Representative

**Professor Earl chaired the committee until her retirement in 2019.*

^1^ Guy’s and St Thomas’ NHS Foundation Trust, London, UK.

^2^ Imperial College London, London, UK.

^3^ Robertson Centre for Biostatistics, University of Glasgow, Glasgow, UK.

^4^ NHS Greater Glasgow and Clyde, Glasgow, UK.

^5^ Department of Oncology, University of Cambridge, Cambridge, UK.

^6^ Patient representative from the UK.

# Recruiting Centres

|  | | Number of Patients Randomized | Percent |  |
| --- | --- | --- | --- | --- |
| Centre Name | |  |  |  |
|  | The James Cook University Hospital, Middlesbrough | 30 | 27.0 |  |
|  | *Darlington Memorial Hospital, Darlington and University Hospital of North Durham, Durham | 17 | 15.3 |  |
|  | Freeman Hospital, Newcastle upon Tyne | 15 | 13.5 |  |
|  | Castle Hill Hospital, Hull | 12 | 10.8 |  |
|  | University Hospital of North Tees, Stockton-on-Tees. | 11 | 9.9 |  |
|  | Derriford Hospital, Plymouth | 6 | 5.4 |  |
|  | Clatterbridge Hospital, Wirral | 5 | 4.5 |  |
|  | Blackpool Victoria Hospital, Blackpool | 5 | 4.5 |  |
|  | Sunderland Royal Hospital, Sunderland | 4 | 3.6 |  |
|  | North Tyneside General Hospital, North Shields | 3 | 2.7 |  |
|  | Kent and Canterbury Hospital, Canterbury | 1 | 0.9 |  |
|  | Weston Park Hospital, Sheffield | 1 | 0.9 |  |
|  | Royal Berkshire Hospital, Reading | 1 | 0.9 |  |
|  | Totals | 111 | 100 |  |

*Both these hospitals are part of the Department of Cancer Services, County Durham and Darlington NHS Foundation Trust.

# Core Laboratories

For the primary endpoint, cTnT was assessed at The Royal Victoria Infirmary, Newcastle upon Tyne Hospitals NHS Foundation Trust, Newcastle-upon-Tyne, UK. Key Staff: Barbara Cresswell, Susan McLellen.

The secondary endpoint cTnI was assessed at the British Heart Foundation Cardiovascular Biomarker Laboratory, Queens Medical Research Institute, University of Edinburgh, Edinburgh, UK. Key Staff: Professor Nicholas Mills, Dr Takeshi Fujisawa.

Echocardiography was performed according to the PROACT echo protocol locally at sites and anonymised images analysed in the echocardiography core laboratory at The James Cook University Hospital, Middlesbrough, UK. Key Staff: Dr Richard Graham, Dr Sharareh Vahabi, Byju Thomas.

# Collaborators

Barbara Cresswell, Royal Victoria Infirmary, Newcastle upon Tyne, UK

Susan McLellen, Royal Victoria Infirmary, Newcastle upon Tyne, UK

Dr Michael Stewart, Consultant Cardiologist, The James Cook University Hospital, Middlesbrough, UK

Professor Andrew Wardley, Consultant Medical Oncologist, London, UK

# Full Inclusion and Exclusion Criteria

Inclusion Criteria

- Adult patients with histopathologically* confirmed breast carcinoma who have received surgery for their breast cancer;
- Planned to receive 6 cycles of EC 90 (total planned dose 540 mg/m2 epirubicin) or FEC 75 (total planned dose 450 mg/m2 epirubicin) adjuvant chemotherapy regimen.

OR

- Adult patients with histopathologically confirmed non-Hodgkin lymphoma planned to receive 6 cycles of R-CHOP or CHOP (total planned dose ≥300mg/m2 doxorubicin) chemotherapy.**

AND

- Written informed consent.

*Patients with HER2+ breast cancer are eligible for inclusion.

** Patients who will receive an alternative anti-CD20 monoclonal antibody (for example O-CHOP) or a combination of rituximab with polatuzumab (e.g. polatuzumab-R-CHP)), are eligible as long as the total planned doxorubicin dose is ≥300mg/m^2^ over 6 cycles

Exclusion Criteria

- Positive baseline cardiac troponin T (≥14ng/L);
- known contraindication to ACE inhibitor e.g. renal artery stenosis, severe aortic stenosis;
- are taking, or have a previous intolerance to ACEI (e.g. angioedema);
- patient already taking other agents acting on the renin-angiotensin-aldosterone system e.g. Aliskiren, angiotensin receptor blockers (ARBs), Entresto (sacubitril/valsartan), spironolactone, eplerenone;
- LVEF <50%*;
- estimated GFR < 30 mL/min/1.73m2 at baseline;
- hyperkalaemia defined as serum potassium ≥5.5mmol/L;
- symptomatic hypotension, or Systolic Blood Pressure <100mmHg;
- poorly-controlled hypertension (Blood Pressure >160/100mmHg**, or ambulatory BP of 150/95mmHg);
- previous myocardial infarction;
- known metastatic breast cancer;
- previous exposure to anthracycline chemotherapy;
- are pregnant or breastfeeding;
- previous Herceptin treatment or planned Herceptin treatment within four weeks following anthracycline chemotherapy;
- for patients of childbearing potential: refusal to use adequate contraception throughout the trial;***
- any other invasive cancer diagnosed and treated in the past 5 years;
- symptomatic or severe asymptomatic radiation-induced cardiac disease;
- participation in other interventional medicinal trials in the past 6 months;
- judgement by the investigator that the patient has a prognosis of < 1 year or are unlikely to complete 6 cycles of chemotherapy;
- judgement by the investigator that the patient is high risk for tumour lysis syndrome (applicable only to NHL patients);
- judgement by the Investigator that the patient should not participate in the study, for example, if the patient is unlikely to comply with study procedures, restrictions, and requirements.

*<50% as defined by Simpson’s biplane method; if absolute measurements are not possible, then a visually normal assessment of LVEF is acceptable for inclusion.

**White coat hypertension is more common, and should be ruled out by an ambulatory blood pressure monitor

***Female patients between the ages of 18 and 50 will receive a pregnancy test at baseline.

# Table 1 Baseline Cancer Sub-types

| Characteristics | | Frequency |  |
| --- | --- | --- | --- |
| Breast Cancer* | |  |  |
|  | Ductal | 58 |  |
|  | Lobular | 8 |  |
|  | Mixed | 1 |  |
|  | Mucinous | 2 |  |
|  | Papillary | 2 |  |
|  | Metaplastic | 2 |  |
|  | Other | 2 |  |
| Non-Hodgkin Lymphoma | |  |  |
|  | Angioimmunoblastic lymphoma | 1 |  |
|  | Diffuse large B cell lymphoma | 36 |  |
|  | Follicular lymphoma | 3 |  |
|  | Peripheral T cell lymphoma | 1 |  |
|  | Other | 1 |  |

*Some patients had more than one cancer type

# Table 2 Serious adverse events

|  | | Enalapril Group | | Standard Care Group | |  |
| --- | --- | --- | --- | --- | --- | --- |
|  | | Number of Events  (n=32) | Number of Patients  (n/N=15/56) | Number of Events  (n=28) | Number of Patients  (n/N=15/55) |  |
| Event Name | |  |  |  |  |  |
|  | Abdominal Pain | 0 | 0 | 1 | 1 |  |
|  | Angioedema | 3 | 1 | 0 | 0 |  |
|  | Chills | 0 | 0 | 1 | 1 |  |
|  | Cough** | 1 | 1 | 0 | 0 |  |
|  | Dehydration | 1 | 1 | 0 | 0 |  |
|  | Dysphagia | 0 | 0 | 1 | 1 |  |
|  | Dyspnoea | 2 | 2 | 2 | 2 |  |
|  | Generalised weakness | 1 | 1 | 0 | 0 |  |
|  | Gastrointestinal mobility issues | 1 | 1 | 0 | 0 |  |
|  | Neutropenia/pancytopenia | 4 | 3 | 4 | 4 |  |
|  | Opiate overdose | 1 | 1 | 0 | 0 |  |
|  | Oral thrush | 1 | 1 | 0 | 0 |  |
|  | Pleuritic pain | 1 | 1 | 0 | 0 |  |
|  | Pyrexia | 1 | 1 | 3 | 3 |  |
|  | Raised CRP | 1 | 1 | 0 | 0 |  |
|  | Raised Troponin I | 0 | 0 | 1 | 1 |  |
|  | Respiratory Tract Infection/Pneumonia | 3 | 2 | 6 | 6 |  |
|  | Rhinorrhea | 1 | 1 | 0 | 0 |  |
|  | Sepsis/Neutropenic Sepsis** | 7 | 7 | 5 | 4 |  |
|  | Sore throat and tongue | 1 | 1 | 0 | 0 |  |
|  | Stroke | 0 | 0 | 1 | 1 |  |
|  | Transient Ischaemic Attack | 1 | 1 | 0 | 0 |  |
|  | Urinary Tract Infection | 0 | 0 | 1 | 1 |  |
|  | Syncope** | 1 | 1 | 0 | 0 |  |
|  | Viral illness | 0 | 0 | 1 | 1 |  |
|  | Vomiting | 1 | 1 | 0 | 0 |  |

*Serious Adverse Events were those events that: resulted in death; were life-threatening; required hospitalisation or prolongation of existing hospitalisation; resulted in persistent or significant disability or incapacity; were a congenital anomaly or birth defect; were any other important medical condition which, although not included in the above which require medical or surgical intervention to prevent one of the outcomes listed.

** 3 Serious Adverse Events were considered by the Principal Investigator at the respective centres to be possibly related to enalapril.

# Table 3 Frequency and grade of all adverse events

|  | | Enalapril Group | Standard Care Group |  |
| --- | --- | --- | --- | --- |
|  | | Number of Events | Number of Events |  |
| Grade | |  |  |  |
|  | 1 | 304 | 172 |  |
|  | 2 | 108 | 75 |  |
|  | 3 | 11 | 11 |  |
|  | 4 | 4 | 0 |  |
|  | Grade missing | 18 | 2 |  |
|  | Total number of events | 445 | 260 |  |
|  | Number of patients reporting events (n/N) | 50/56 | 45/55 |  |

# Table 4 Adverse events (adverse reactions) considered to be related to enalapril

|  | | Number of Events  (n=37) | Number of Patients  (n/N=21/56) |  |
| --- | --- | --- | --- | --- |
| Event Name | |  |  |  |
|  | Blurred vision | 1 | 1 |  |
|  | Cough | 7 | 7 |  |
|  | Decreased appetite | 1 | 1 |  |
|  | Dry throat | 1 | 1 |  |
|  | Dizziness | 9 | 8 |  |
|  | Dry mouth | 1 | 1 |  |
|  | Headache | 3 | 2 |  |
|  | Hypotension | 4 | 4 |  |
|  | Light headedness | 2 | 2 |  |
|  | Malaise | 1 | 1 |  |
|  | Nausea | 5 | 4 |  |
|  | Taste changes | 1 | 1 |  |
|  | Vomiting | 1 | 1 |  |

Figure 1 Mean Blood Pressure (95% CI) by randomisation group
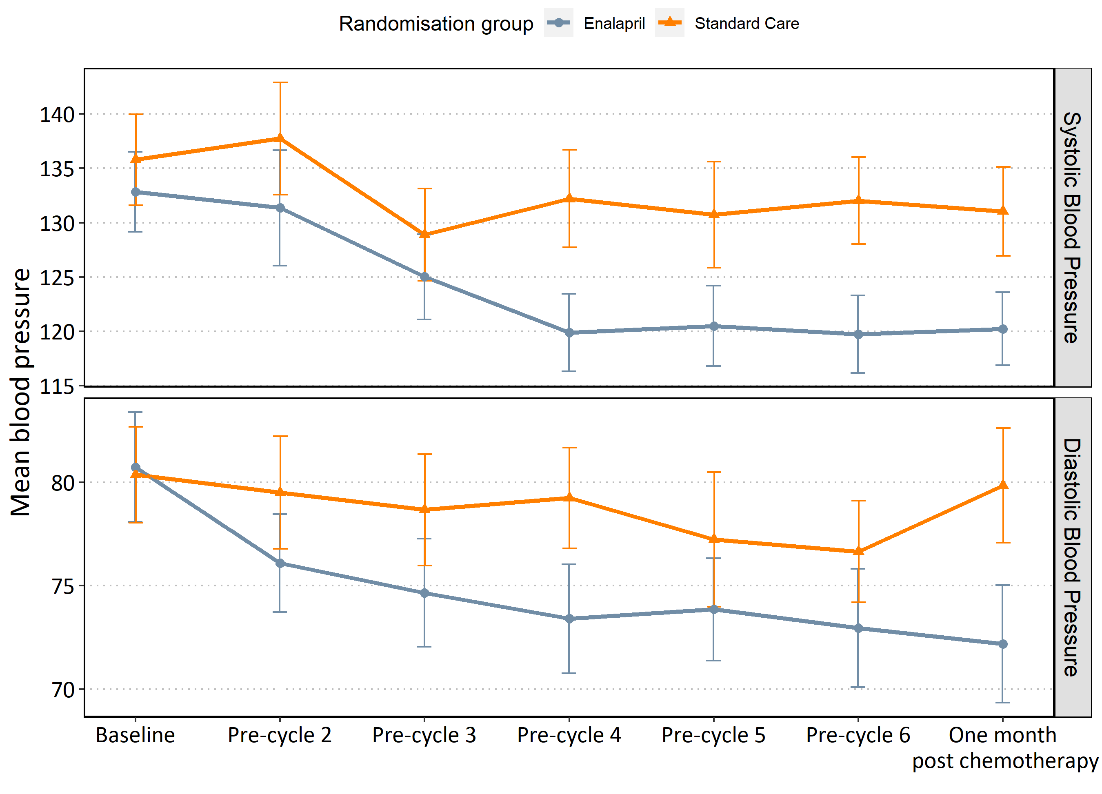


# Figure 2 Intention-To-Treat, Per-Protocol and As-Treated analyses of the primary outcome


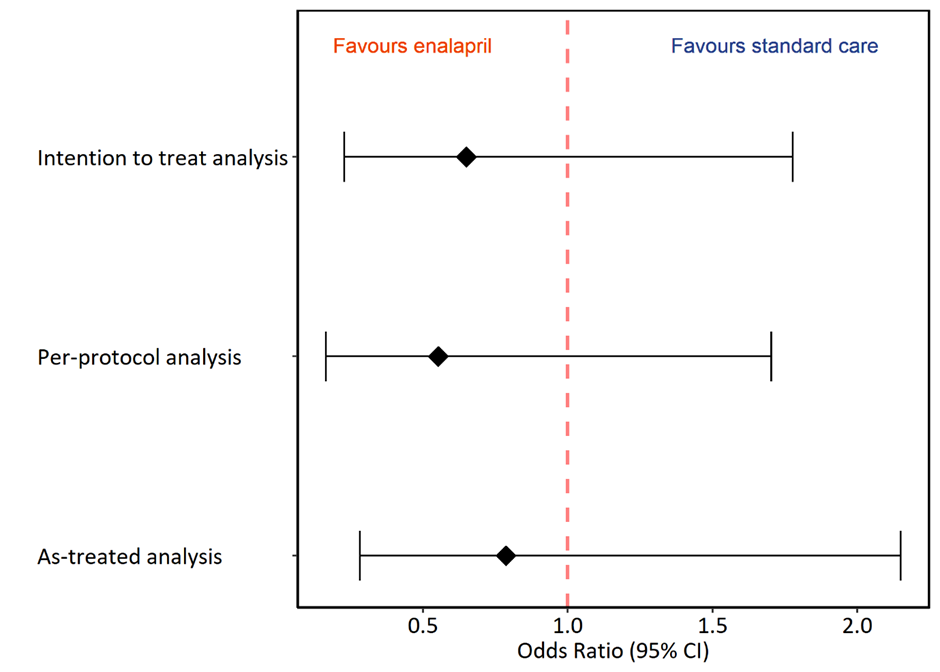


Figure 1 is a forest plot showing the Intention-To-Treat, Per-Protocol and As-Treated analyses of the primary outcome. The diamond represents the odds ratio, with figures below 1.0 indicating the intervention is favoured. Confidence intervals are show as bars extending from the diamonds; those crossing 1 are indicative of no statistically significant effect.

# Figure 3. Forest plot of regression results for primary outcome by intervention group and other baseline characteristics


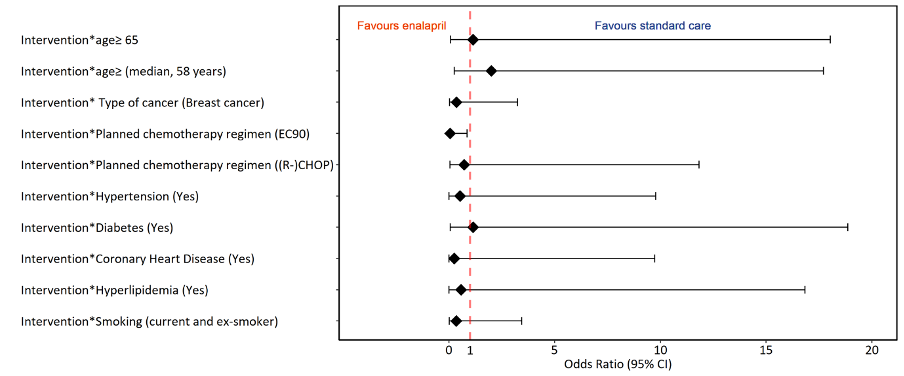


Reference: age<65, age< (median, 58 years), non-Hodgkin Lymphoma, Planned chemotherapy regimen (FEC75), no hypertension, non-diabetic, no coronary heart disease, no hyperlipidemia, non-smoker. The diamond represents the odds ratio, with figures below 1.0 indicating the intervention is favoured. Confidence intervals are show as bars extending from the diamonds; those crossing 1 are indicative of no statistically significant effect.
